# *The Organon* and Materia Medica Club of the Bay Cities of California

**Published:** 1896-04

**Authors:** W. E. Ledyard

**Affiliations:** Secretary


### *THE ORGANON* AND MATERIA MEDICA CLUB OF THE BAY CITIES OF CALIFORNIA.

The regular semi-monthly meeting was held at the office of Dr. J. M. Selfridge, in Oakland, Friday evening, October 18th, 1895.

Members present: Drs. J. M. Selfridge, S. E. Chapman, W. E. Ledyard, M. T. Wilson, E. W. Bradley, G. J. Auger. Visitors: Dr. T. C. Coxhead and Mr. A. Johnson.

The meeting was called to order at 8.15 o'clock by the President, Dr. J. M. Selfridge.

The minutes of the previous meeting were read by the Secretary, Dr. W. E. Ledyard, and after slight corrections by Drs. Selfridge and Ledyard, were approved.

The President then asked the Secretary for the resolutions upon the death of Dr. James E. Lilienthal, which he had been instructed to prepare. The Secretary reported that he had not yet prepared the resolutions, whereupon the President ordered them done for the next meeting.

Dr. Chapman then presented a paper entitled, "What is Truth?" Dr. Wilson moved that the sentiments expressed in the paper be indorsed by the Club; seconded by Dr. Ledyard, and unanimously carried.

Dr. Selfridge commenced the reading of *The Organon* at Section 281.

### DISCUSSION.

Dr. Selfridge—Disease renders the functions painful. As in eye troubles, the eyes are more sensitive to light. Light to the healthy eye is all right, but to a diseased eye it gives pain.

Dr. Ledyard—As of one with a cut on the finger; he supposes he is striking it all of the time when it is simply that the finger is more sensitive.

Sections 282 and 283 were read.

DISCUSSION.

Dr. Chapman—There is one thing in regard to the administration of the high potencies: Even if not really indicated by every symptom, if similar at all, they will do good.

Dr. Selfridge—So far as they are similar, they will do good.

Dr. Wilson—The question of the smallness of the dose is perplexing. It requires much skill on the part of the physician to know when to use the high or low potencies.

Dr. Chapman—Right at this point I take issue in regard to the shaking process to obtain the high potencies. Take, for instance, a solution of salt, a few grains in an ounce of water. You can shake it for a thousand years and it will never produce the result you would get from the 200th potency.

Dr. Selfridge then read note 270 to explain this.

Dr. Chapman—Would you take an ounce of the tincture of Opium and shake it, and then expect to get from it the effect of the 200th potency?

Dr. Auger—Hahnemann means a minimum quantity of the drug to a maximum quantity of water.

Dr. Selfridge—If you take an ounce of Opium you would have to take many ounces of water.

Dr. Wilson—Did not we read a section at our last meeting stating that Hahnemann went through a process of vibrations or shaking until he obtained the 30th potency?

Dr. Bradley—How did he know he had the 30th potency?

Dr. Wilson—By the effect of the medicine upon the patient.

Dr. Bradley—I do not believe he could do it.

Dr. Selfridge—It cured just as well as the 30th potency would do. In other words, he broke half a grain of soda into molecules or atoms.

Dr. Auger—He does not advocate that method of potentization.

Dr. Bradley—I think the efficacy of the drug all right; but when it comes to potentization I think he was mistaken.

Dr. Chapman—It is no wonder that Hahnemann would make some mistakes, and I do believe he was mistaken here.

Dr. Selfridge—You have never tried it.

Dr. Chapman—Never.

Dr. Selfridge—For all I know, what Hahnemann says here may be true of all medicines.

Dr. Chapman—Then let us get at it and we will convert all the allopaths.

Dr. Ledyard—I think he states in a note that succussion makes the drug very dangerous; and this is not so with our high potencies.

Dr. Selfridge—I do not think he says "*dangerous.*"

Section 284 was read.

DISCUSSION.

Dr. Chapman—Some substances cannot be broken up so minutely. Silicea cannot be divided by manufacturers. A gentle process cannot break this hard substance.

Dr. Selfridge—The particles are already broken up and are divisible by water.

Dr. Auger—Dr. Selfridge gave a good illustration of this in his paper, "Infinitesimals in Nature," when he spoke of the dog being able to trace his master through smelling the particles from the sole of his master's shoe; they must have been exceedingly minute, and especially after the man had traveled miles; and yet these small particles must be in existence.

Dr. Chapman—It is simply an emanation or effluvia which possibly contains no substance at all.

Dr. Selfridge—This is an assumption. You go back to Silicea. They must reach a point in the trituration of the original drug, if it is not soluble, where they can divide it.

Dr. Ledyard—The proof is that the very high potencies of *Silicea* cure better than lower ones.

Dr. Selfridge—You must set free the molecules and atoms before you can do anything with them.

Dr. Chapman—Take for instance *Luna,* the moon; if this can be bottled and will cure, you certainly will admit that there is no material in it. Can you imagine matter in the one-hundreth thousandth potency after it slops over the pan in preparing?

Dr. Selfridge—Bottled Luna is material. Science teaches us that light is material. If light is not matter then science is at fault.

Dr. Chapman—Science be hanged.

Section 285 was read.

DISCUSSION.

Dr. Chapman—Hahnemann seems to lay great stress upon the dose of pellets, etc. I do not believe in this. I think one pellet is as good as a thousand.

Dr. Selfridge—I agree with you on that point.

Section 286 was read.

DISCUSSION.

Dr. Auger—This rather bears Dr. Selfridge out in his explanation. If it was spirit, that amount of fluid would be useless. Here it says, "*the greater the amount of fluid, the more it divides it.*"

Section 287 was read.

DISCUSSION.

Dr. Selfridge—We generally believe that the effect of high degrees is more lasting. Here he says the opposite.

Dr. Chapman—That is certainly a mistake.

Dr. Ledyard—It must be a mistake in translation.

Dr. Wilson—We undoubtedly believe the opposite to what is said here.

Section 288 was read.

DISCUSSION.

Dr. Wilson—This note is a contradiction to the one we just read. Here we have it by olfaction, and that he claims to be stronger.

Dr. Bradley—Are there any members here who practice olfaction ?

Unanimous reply ; *No.*

Dr. Bradley—Some drugs might produce medicinal effects by this method, as Nitrite of Amyl.

Dr. Selfridge—I will not say that it is not as Hahnemann says until I have tried it. Many times I have disbelieved statements made by Hahnemann, and tried them and found that he was right.

Dr. Chapman—Constantine Hering gives a case of puerperal convulsions which he cured by holding pellets of Opium to the nose.

Dr. Wilson—Olfaction may be good where there is clenched teeth.

Sections 289–292 were read.

### Discussion.

Dr. Selfridge—Speaking of taking up the material substance into the system, especially Mercury. I know of an instance related by a Professor of Materia Medica: Maiden lady in the habit of taking Blue-mass for everything. After a time she could tell when it was going to storm. Finally she could not cure herself with the Blue-mass and then went to him. He sent her to Dr. Foster's sanitarium (allopathic), for the water-cure. She had packs, sweats, and baths all winter. In the month of May she walked out to a maple grove about one hundred rods from the sanitarium, where there was a Sulphur spring. She perspired freely, and finally her face and hands turned black. She ran back to the sanitarium. The Doctor said that the Mercury in the skin had united with the Sulphur of the Sulphuretted Hydrogen. He told her to take a bath and she was cured.

Dr. Wilson—I have a case that I would like to relate. About a week ago a young man about eighteen or nineteen years of age came to me. He had been taking preparations from the drug store for his bowels, which he said were running off very freely. He had been to see Dr. D——, an allopathic physician, who had given him an opiate, I suppose. His temperature was from 100° to 102⅓°; pulse full and strong; tongue swollen, dirty coating, red tip; some nausea; no vomiting; no straining; the bowels were not running now as he was under the influence of the medicine; cough dry and splitting, with a breaking of white mucus which would leave a sore, scratching feeling ; no tympanitis ; bowels flaccid ; little pain. I told the mother *if the bowel trouble commenced again not to be worried.* Soon it came again, with every hour a watery, green stool ; no slime ; urine free ; could not tell the nature of the urine ; restless and tossing about ; thirst for water often, but a little at a time ; no appetite ; had been taking only milk for two or three days ; temperature remained the same ; he did not sleep much at night. What was he threatened with ?

Dr. Selfridge—It would be almost impossible to tell on account of the drugs given.

Dr. Chapman—I should think bilious or typhoid fever.

Dr. Wilson—I gave him *Arsenicum*$^{200}$. No change. Next morning I repeated the remedy. He wakes up at one or two o'clock, then goes into a profuse perspiration, falls asleep and wakes up again in the morning. I gave to-day one dose of *Calcarea*$^{200}$ for the perspiration. I thought it was threatening typhoid. Dr. D—— said he was threatened with cholera. I do not see any reason for such a diagnosis.

This ended the discussions and the President than announced that at the next meeting *The Organon* would be commenced at Section 1.

Dr. Wilson moved that the next meeting be held at the office of Dr. George H. Martin, 606 Sutter Street, San Francisco, the first Friday in November. Seconded by Drs. Ledyard and Chapman, and carried.

The President then declared the meeting adjourned.

W. E. LEDYARD, *Secretary.*

Reported by Eleanor F. Martin, M. D.

---

